# Application of Quantitative PCR in the Diagnosis and Evaluating Treatment Efficacy of Leishmaniasis

**DOI:** 10.3389/fcimb.2020.581639

**Published:** 2020-10-07

**Authors:** Yun Wu, Xiaojun Tian, Nan Song, Minjun Huang, Zhaoyong Wu, Shaogang Li, Nicholas R. Waterfield, Bin Zhan, Lei Wang, Guowei Yang

**Affiliations:** ^1^Emergency and Critical Care Center, Beijing Friendship Hospital, Capital Medical University, Beijing, China; ^2^Beijing Institute of Tropical Medicine, Beijing, China; ^3^Warwick Medical School, Warwick University, Coventry, United Kingdom; ^4^Department of Pediatrics and National School of Tropical Medicine, Baylor College of Medicine, Houston, TX, United States

**Keywords:** human leishmaniasis, quantitative PCR, diagnosis, treatment efficacy, *L. donovani*, *L. infantum*, *L. major*

## Abstract

Leishmaniasis is still a serious neglected tropical disease that may cause death in infected individuals. At present, the clinical diagnosis and treatment monitoring still rely on parasitological culture and microscopy that needs experienced technicians. The low sensitivity and inconvenience of microscopic examination could cause misdiagnosis and relapse of leishmaniasis. There is an urgent need for developing a sensitive and easily operated diagnostic method for the diagnosis and disease management of leishmaniasis. Thus, a quantitative real-time PCR (qPCR) based on the conversed regions of kinetoplast minicircle DNA (mkDNA) of *Leishmania spp*. was developed to detect different species of *Leishmania*. The designed mkDNA-based qPCR was able to detect as low as one copy of *Leishmania* mkDNA or DNA from single parasite. It also detected Pan-*Leishmania* protozoa including *Leishmania donovani, Leishmania infantum* and *Leishmania major* without cross-reaction with other pathogen DNAs available in our lab. This method was clinically applied to quantitatively detect skin lesion samples from 20 cutaneous leishmaniasis (CL) and bone marrow and/or PBMC samples from 30 current and cured visceral leishmaniasis (VL) patients, and blood samples from 11 patients with other infections and 5 normal donors as well. Total 20 skin lesion samples from current CL patients and 20 bone marrow and/or PBMC samples from current VL patients were all detected as positive with qPCR without cross-reaction with samples from patients with malaria, brucellosis and dengue or normal donors. Two VL patients with parasite converted to microscopically negative after treatment were detected positive with qPCR. The patients with bigger skin lesion in CL and higher level of immunoglobulin or splenomegaly in VL, had the higher parasite load detected by qPCR. The parasite load was significantly reduced after treatment. In conclusion, the mkDNA-based qPCR assay that we developed in this study can be used not only for diagnosis of both cutaneous and visceral leishmaniasis with high sensitivity and specificity, but also for evaluating the severity and treatment efficacy of this disease, presenting a rapid and accurate tool for clinical surveillance, treatment monitoring and the end point determination of leishmaniasis.

## Introduction

Leishmaniasis is a serious neglected tropical disease caused by the protozoan parasite *Leishmania. spp*. transmitted by the bite of infected female phlebotomine sandflies (Burza et al., 2018). It has been estimated that there are 700,000–1 million cases of human infection with ~26,000–65,000 death reported annually (Burza et al., 2018). The reported cases are only small fraction of the real infection and many of them are neglected or misdiagnosed as other diseases, in addition to more than 350 million people living under high infection risk (Ghorbani and Farhoudi, 2018). The infection of *Leishmania spp*. causes a spectrum of diseases, including cutaneous leishmaniasis (CL), visceral leishmaniasis (VL), and mucocutaneous leishmaniasis (MCL) (Torres-Guerrero et al., 2017), with CL and VL representing the most common forms. Globally, it has been estimated that ~90% of CL cases are distributed in 10 worst affected countries including Afghanistan, Algeria, Colombia, Brazil, Iran, Syria, Ethiopia, North Sudan, Costa Rica, and Peru (Bailey et al., 2017). The *Leishmania* parasites causing CL can be divided into Old World species *Leishmania major, Leishmania tropica, Leishmania aethiopica*, and the New World species *Leishmania amazonensis, Leishmania mexicana, Leishmania braziliensis*, and *Leishmania guyanensis* (de Vries et al., 2015). The symptoms of CL usually present as nodule, patch/plaque and ulcerative skin lesions. Generally, CL is not life-threatening, however the parasite can spread through the lymphatic tissues, leading to mucocutaneous leishmaniasis or so-called “diffuse CL” that could result in extensive midfacial destruction and stigma (de Vries et al., 2015). Visceral leishmaniasis (VL), also known as kala-azar, is the most severe form of leishmaniasis. VL is mostly endemic in seven countries including India, Brazil, Ethiopia, Kenya, Somalia, Sudan, and South Sudan (Bi et al., 2018). The main etiological agents of VL are *Leishmania donovani* and *Leishmania infantum* (Bi et al., 2018). The main clinical presentations of VL include irregular fever at onset, followed by splenomegaly pancytopenia, hepatomegaly, hypergammaglobulinaemia and a permanent loss of weight. Without effective treatment the disease exacerbates by 2 years, typically with fatal consequences. In China, visceral leishmaniasis caused by *L. donovani* used to be seriously endemic in the central China. It has been eliminated in most endemic regions in 1960s since national control programs were launched in 1950s (Guan, 2009; Yang et al., 2014). However, some sporadic cases are still reported in the western part of China (Coordinating Office of the National Survey on the Important Human Parasitic Diseases, 2005) with two types of visceral leishmaniasis caused by *L donovani* transmitted by peridomestic *Phlebotomus longiductus* or by *L. infantum* transmitted by *Phlebotomus chinensis* (Wang et al., 2012). In recent years, leishmaniasis become re-emerging as more imported cases of both visceral and cutaneous leishmaniasis were reported in the major cities along east coast of China due to the increasing travel and business activities with African and other developing countries, which has brought more attention and concerns to physicians regarding the clinical diagnosis and treatment of this neglected tropical disease (Wang et al., 2017, 2019).

In current clinical practice, definitive diagnosis of leishmaniasis relies on a comprehensive analysis of the clinical manifestation, travel history and epidemiology, along with time/labor consuming laboratory tests (Aronson et al., 2016). Misdiagnosis of leishmania to other infection diseases is common due to the unspecific clinical manifestations especially for VL. The parasitological examination for the presence of the amastigote stage of the protozoa in the tissues of patients is considered as gold standard for the definitive diagnosis of leishmaniasis. However, it requires experienced and trained parasitologists or technicians, and the sensitivity of the parasitological examination of biopsy specimens by microscopy ranges only between 60 and 95% for cases of VLs and between 78.3 and 90.4% for cases of CL (Aronson et al., 2016; de Morais et al., 2016; Galluzzi et al., 2018). Furthermore, parasite can be identified by *in vitro* culture only in 39% VL samples and in 64.3% CL samples (Bahrami et al., 2018). Previous studies have shown that *Leishmania* antigens can be detected in urine, however both the sensitivity (28–82%) and specificity (49–53%) of this method were unacceptably low (Galluzzi et al., 2018; Abeijon et al., 2019; Mondal et al., 2019). An alternative immunological based assay is to detect antibody anti Leishmania rK39 antigen with sensitivity of 67–100% (Pagliano et al., 2016; van Griensven and Diro, 2019), however, this immunological assay is unable to differentiate between current and historical infection (Saliba et al., 2019). Regular PCR is sensitive, but cannot be used to monitor the parasite load during medical treatment, and so is not appropriate for ongoing patient/disease management, or evaluating the treatment efficacy (Mesa et al., 2020). Therefore, it is urgently needed to develop a diagnostic method with high sensitivity and quantitative measurement of infected parasite for clinical physicians.

In recent years molecular diagnostic tools for various infections have been developed for clinical application. These include PCR-based assays, which are recognized as rapid, sensitive method with ability to reliably discriminate between virus, bacteria, fungi, and parasites. We present here a new diagnostic method for leishmaniasis which uses quantitative real-time PCR (qPCR) technology to specifically detect *Leishmania* kinetoplast minicircle DNA (mkDNA). The mkDNA exhibits species-specific sequence divergence and therefore can be used as a marker for developing DNA probe-based diagnostic test for leishmaniasis and identification of *Leishmania* species that causes the disease (Spithill and Grumont, 1984; Rogers and Wirth, 1987). The mkDNA-based qPCR we developed in this study showed higher sensitivity than conventional parasite examination and high specificity for leishmaniasis. Except for its diagnostic purpose, it also showed the advantage as a tool to determine the disease severity and to evaluate the treatment efficacy and prognosis of the disease.

## Methods

### Ethics

This project was approved by the Ethics Committee of Beijing Friendship Hospital (Beijing, China) with approval number of 2020-P2-005-01. Informed consents were obtained from all involved patients.

### Patients and Samples

The whole blood, bone marrow samples were collected from 23 clinically diagnosed current VL patients and 7 cured VL patients (with no parasite identified) at Beijing Friendship Hospital, Capital Medical University from July 2015 to Jan. 2020. The current VL patients presented with symptoms such as persistent fever, splenomegaly and/or hepatomegaly, positive in rK39 rapid diagnostic tests (RDT) and *Leishmania* amastigotes found in their blood and/or bone marrow samples under microscope. Skin lesion samples were obtained from 20 diagnosed CL patients. All patients' skin lesion appeared as ulcer and nodule/plaques features in which *Leishmania* amastigotes were identified. All tissue samples including skin lesion autopsy from CL patients, PBMC and bone marrow from VL patients were stored at liquid nitrogen till use.

In addition, 11 human blood samples were taken from patients with other infections including five with malaria, five with brucellosis, and one with dengue fever as non-leishmaniasis controls. Blood from five healthy volunteers were also included as normal control.

### Primer and Probe Design

Total 25 mkDNA sequences from different species of *Leishmania* parasite were collected from GenBank and aligned using BIOEDIT software (v7.0.1, Ibis Biosciences, Carlsbad, CA, USA). Primers and probes were designed based on the conserved region of sequence shared by four common pathogenic species of *L. donovani, L. infantum, L. major*, and *L. tropica* using Primer Express 3.0.

### DNA Extraction

DNA was extracted from cell and tissue samples using a TIANGEN DNA extraction kit (TIANGEN, DP705, Beijing, CHN) according to manufacturer's instructions. Briefly, about 20 mg lesion tissue or 200 μl of bone marrow or PBMC were digested with 20 μl proteinase K in 300 μl buffer GHA for 3 h at 56°C on a plate shaker (1,500 rpm), 300 μl lysis buffer GHL and 300 μl isopropyl alcohol were added and vortexed. The treated tissue samples were then mixed with magnetic beads in the kit to bind the DNA. The DNA-bound beads were subsequently washed and dried for 15 min at room temperature. Finally, 100 μl elution buffer was added and incubated at 56°C for 10 min to release the DNA from the magnetic beads. The beads were removed by centrifugation and the DNA containing supernatants were stored at −20°C till use.

### Positive Control Plasmid Construction

The mkDNA target sequence fragment was PCR amplified using the primers designed as above and purified with DNA purification kit (TIANGEN, DP214, Beijing, CHN). The amplified mkDNA fragment was ligated into plasmid pUC19 (TAKARA, 3219, Tokyo, Japan) using *EcoR*I and *Hind*III sites. The correct insert of target DNA in recombinant plasmid DNA was confirmed by PCR amplification and DNA sequencing.

### Quantitative Real-Time PCR Assay

All tests were conducted using the Applied Biosystems 7500 Fast Real-Time PCR System (ABI) in 10 μl reaction volume. The reaction mixtures contained the following; 5 μl of Promega GoTaq® Probe qPCR Master Mix (Promega, A6101, Madison, WI, USA), 200 nM Forward primer KD3, 200 nM Reverse primer KD4, 100 nM hydrolysis probes (5′FAM/3′TAMRA), CXR reference dye (30 nM), and 1 μl of template DNA (5–50 ng). The following cycle conditions were used; 95°C for 2 min followed by 40 cycles of 95°C for 15 s, 60°C for 30 s. A water only negative control (NTC-H_2_O) and a positive control using the mkDNA/pUC19 plasmid DNA were included in all experiments.

### Sensitivity and Specificity Analysis

#### Analytical Sensitivity

The limit of detection (LOD) for the plasmid template was defined as the lowest number of detectable copies based on repeated tests (*n* = 3), using 1,000, 100, 10, 5, and 1 plasmid copies/reaction and the working assay precision (intra-assay SD <0.5 and inter-assay CV <5%). For testing the LOD for the parasite, cultured *L. infantum* promastigotes were enumerated by counting under a microscope. The *Leishmania* parasite suspension was then diluted in blood from healthy volunteer as 1, 5, 10, 50, 100 parasites in 200 μl blood and the total DNA was extracted from each dilution. The functional sensitivity was defined from the experimentally derived assay precision measurement (intra-assay SD <0.5 and inter-assay CV <5%). The sensitivity of qPCR was compared with the sensitivity of clinical diagnosis based on the typical clinical manifestation and parasite determination under microscope.

#### Specificity

The genomic DNA samples previously extracted from *Plasmodium falciparum, Toxoplasma gondii, Rickettsia tsutsugamushi, Mycobacterium leprae*, and *Brucella melitensis*, including DNAs extracted from 11 patients with other infections (mentioned above) were used to test the specificity of our developed *Leishmania* PCR/qPCR assay.

#### Clinical Sensitivity, Specificity, and Monitoring Performance of qPCR

Total 66 clinical samples including 20 skin lesion tissues from CL patients, 30 PBMC/bone marrow samples from VL patients, 11 blood samples from patients with other infections, and five normal donors, were analyzed using the designed qPCR assay for evaluating its sensitivity and specificity for leishmaniasis.

### Statistical Analysis

The qPCR data analysis was performed using the ABI 7500 software 2.3. Statistical analysis was carried out using the SPSS software version 20.0 and visualized on Graphpad Prism version 5.0. The Mann-Whitney *U*-test was used to statistically compare between groups. *P* < 0.05 was considered as statistical significance.

## Results

### Design of a Quantitative Real-Time PCR Assay for Pan-*Leishmania* Detection

**Figure 1:**
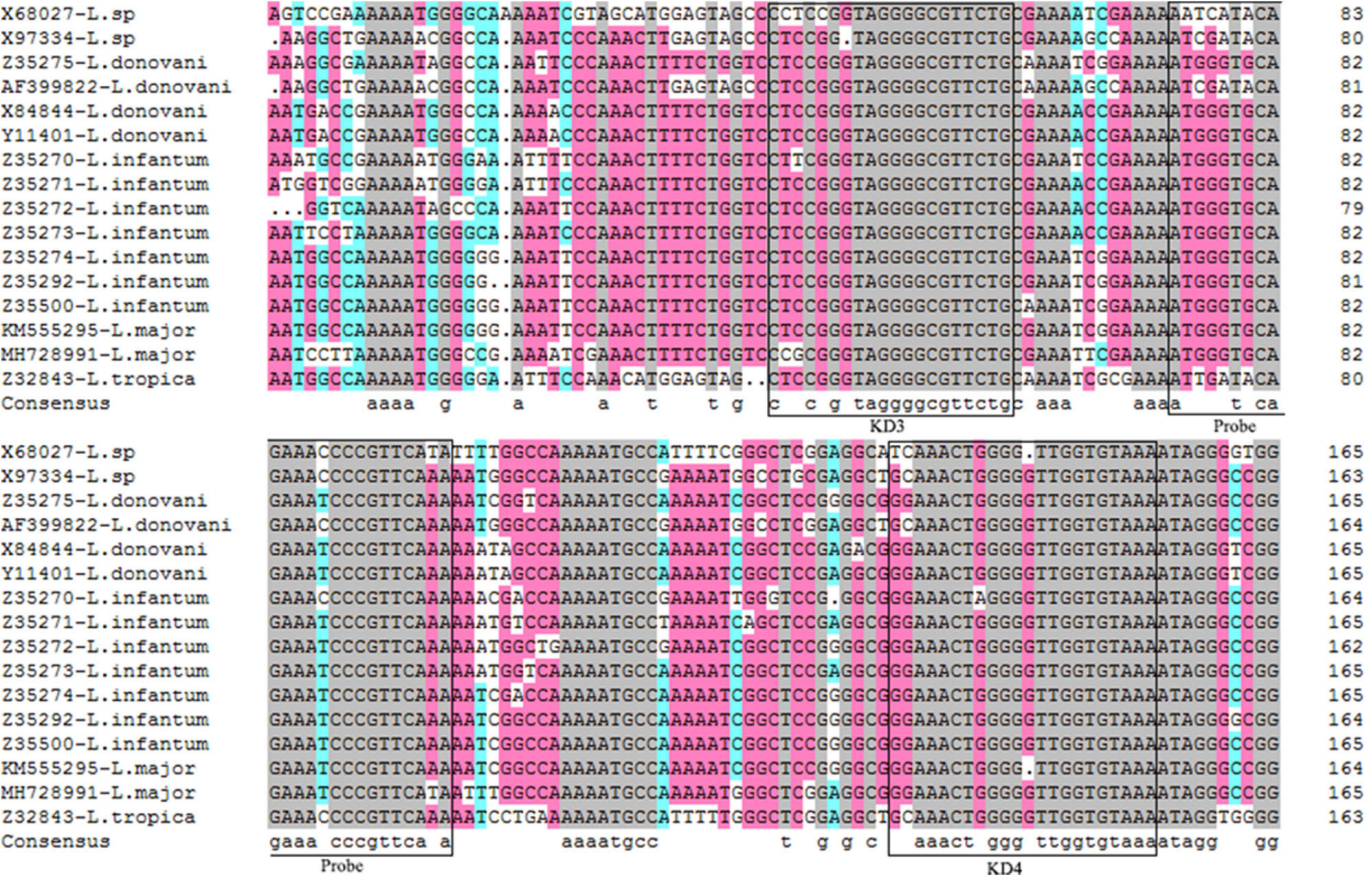
Alignments of mkDNA sequences from major *Leishmania* species. Sequences were aligned using CLUSTALW and prepared for display using BOXSHADE. Identical amino acids are shaded with different color. The consensus sequences were squared for making forward primer KD3 and reverse primer KD4, and probe. The gray mark represents the sequence homology 100%, the pink mark represents the sequence homology ≥75%, the blue mark represents the sequence homology ≥50%.

Alignments of mkDNA sequences from major *Leishmania* species reveals that mkDNAs of *L. donovani, L. infantum, L. major*, and *L. tropica* share sequence identity of 89.67%. Three regions of mkDNA sequence are conserved in all four species of *Leishmania* with consensus sequence of CSB-I (GGGCGT), CSB-II (CCCCGTTC), and CSB-III (GGGGTTGGTGTA). Primers were designed based on these consensus regions as forward KD3: 5′-TCCGGGTAGGGGCGTTCTG-3′ and reverse KD4: 5′-TTTACACCAACCCCCAGTTTCC-3′. A fragment DNA sequence within the amplified region was chosen for synthesizing probe: 5′-FAM-TTTGAACGGGATTTCTGCACCCAT-TAMRA-3′ for qPCR detection (Figure 1).

**Figure 2:**
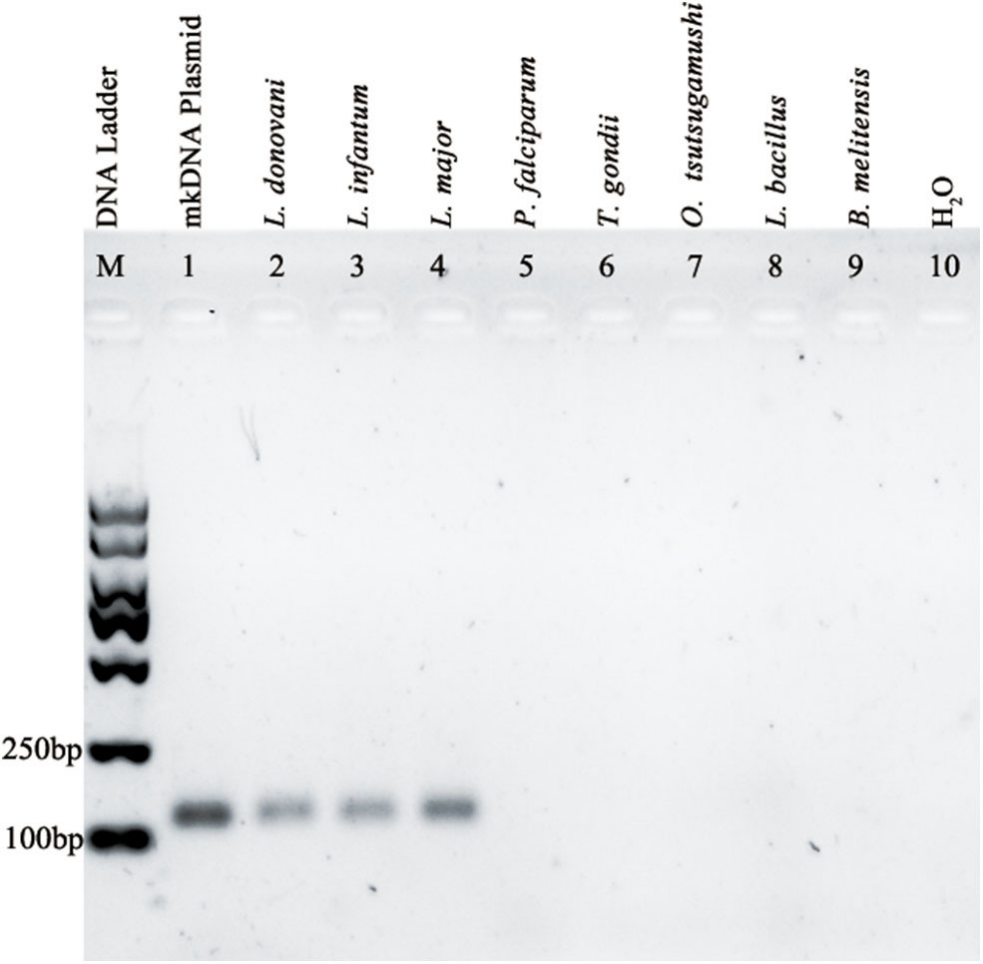
Amplification of *Leishmania* specific mkDNA in *L. donovani, L. infantum*, and *L. major* using mkDNA-based PCR method without cross-reaction with *P. falciparum, T. gondii, R. tsutsugamushi, M. leprae, and B. melitensis*.

A PCR assay using this pair of primers was performed to detect DNA samples extracted from cultured *L. donovani* promastigote or from samples of patients infected with *L. infantum* or *L. major*. The results showed that the PCR based on the *Leishmania* mkDNA conserved sequence was able to amplify a 114 bp fragment from both visceral (*L. donovani, L. infantum*) and cutaneous *Leishmania* spp (*L. major*). However, these primers didn't recognize any DNA from samples of *P. falciparum, T. gondii, R. tsutsugamushi, M. leprae, and B. melitensis* (Figure 2).

### Establishment of qPCR Assay for Testing *Leishmania* mkDNA

**Figure 3:**
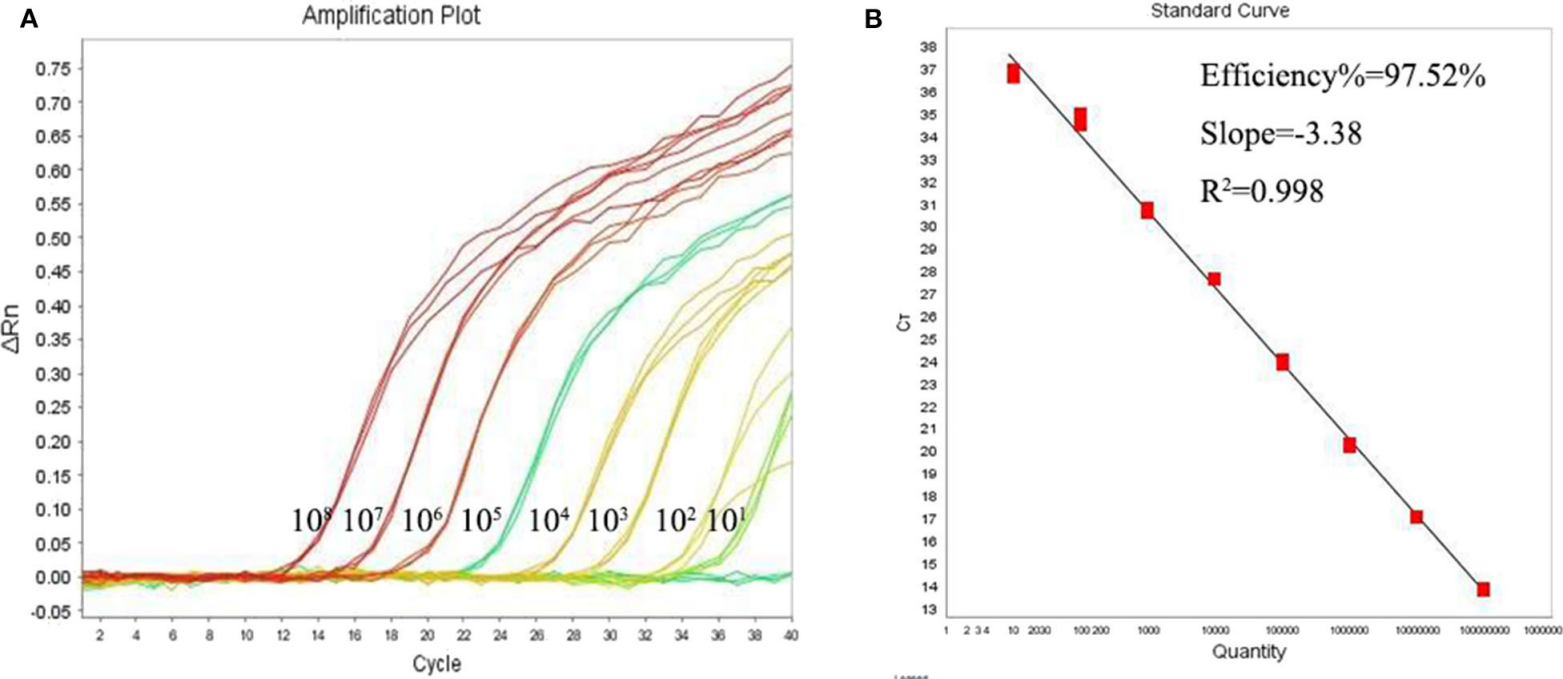
Quantitative correlation between mkDNA copy number and threshold cycle of the *Leishmania* qPCR assay. **(A)** *Leishmania* mkDNA plasmid was diluted in serial from 10^1^ to 10^8^ copies/reaction and subjected to qPCR. ΔRn = Rn (normalized reporter)—baseline. **(B)** Linear regression of Cq vs. lg copy number of mkDNA plasmid. Ct, Cycle threshold.

Using the primers and probe described above, we established a qPCR assay which was able to detect as little as single copy of *Leishmania* mkDNA plasmid or DNA from single *Leishmania* parasite with CV <5.0% (Table 1). Our results showed that the standard curve generated using serial copy number of plasmid DNA was linear over an 8-log range with a correlation coefficient (*R*^2^) of 0.998 (Figures 3A,B).

**Table 1:** Assessment of analytical sensitivity of qPCR assay to detect *Leishmania* mkDNA and parasite in blood.

**Sample type**	**Concentration**	**Mean Cq (standard deviation)**	Inter-assay* CV (%)
Plasmid (copies/reaction)	1,000	30.77 (0.10)	0.86
	100	34.71 (0.23)	1.28
	10	36.80 (0.16)	1.46
	5	37.01 (0.48)	2.1
	1	37.89 (>0.5)	>5
	0	undetected	-
Parasite in whole blood (parasite/reaction)	100	17.93 (0.32)	3.9
	50	19.22 (0.09)	0.68
	10	22.25 (0.22)	0.64
	5	23.24 (0.34)	0.90
	1	26.31 (0.06)	0.96
	0	undetected	-

*CV, Coefficient of Variation*.

* *The inter-assay was evaluated based on triplicate reaction*.

The intra assay and inter assay CV of Ct values for the 20 replicates was <2%, indicating a high precision in the assay (Table 2). Taken together, our results show that this qPCR assay is established with high sensitivity and precision. The qPCR was further used to test clinical specimens.

**Table 2:** Precision of intra and inter-assay of mkDNA-based qPCR assay.

**Sample ID**	***N***	**Intra-assay**			***N***	**Inter-assay**		
		**Mean Cq**	**SD**	**CV (%)**		**Mean Cq**	**SD**	**CV (%)**
1	20	19.04	0.24	1.2	20	18.90	0.33	1.7
2	20	27.53	0.20	0.7	20	26.98	0.37	1.3

*N, number of samples; SD, standard deviation; CV, Coefficient of Variation*.

### Sensitivity and Specificity of qPCR for Detecting *Leishmania* Parasite in Patients With Clinically Diagnosed Leishmaniasis

To test the sensitivity and specificity of established mkDNA-based qPCR to diagnose leishmaniasis, total DNAs were extracted from specimens from 50 patients with clinically diagnosed leishmaniasis including 20 CL and 30 VL. Among the 20 patients of CL, all are current patients with skin lesion and parasite identified under microscope. Among the 30 patients with VL, parasites have been identified in 21 patients either in blood or in bone marrow through direct microscopy examination or parasite culturing *in vitro*, nine patients were previously diagnosed as leishmaniasis by parasite examination and currently determined as cured without parasite identified in microscopic examination. After being tested by qPCR, the skin lesion samples from 20 CL patients were all positive. Among the 21-parasite confirmed VL patients, 20 were positive with qPCR, one with parasite identified only in the bone marrow (not in blood) was negative with qPCR for detecting blood sample. For other nine clinically cured VL patients (without parasite detected in blood and bone marrow), two of them were detected as positive in blood samples by qPCR (Table 3). The results indicate that the developed qPCR assay is sensitive (sensitivity 95.6%, 22/23) and the clinical diagnosis should combine the results of parasite detection in blood or bone marrow with qPCR test. Even if parasite examination converts to negative, it is still possible the trace parasite or its disrupted DNA could be detected using qPCR. The total sensitivity and diagnostic accuracy for all leishmaniosis can reach up to 97.6% (42/43) and 98.5% (65/66) (Table 3). To evaluate the specificity of qPCR for detecting *Leishmania*, DNAs were extracted from blood samples of 11 patients with infection of other pathogens including five with *P. falciparum* (malaria), five with *B. melitensis* (brucellosis) and one with Dengue virus, and five healthy normal people. All samples were detected as negative with the qPCR assay, indicating mkDNA-based qPCR has no cross reaction with other pathogens with 100% specificity for detecting *Leishmania* infections (Table 3).

**Table 3:** Sensitivity and specificity of parasite detection and qPCR to diagnose cutaneous leishmaniasis (CL) and visceral leishmaniasis (VL).

**Type**	**PCR Sample**	**Parasite+/qPCR+**	**Parasite–/qPCR+**	**Parasite+/qPCR–**	**Parasite–/qPCR– (Recovered)**	**Parasite/qPCR Sensitivity%**	**Specificity%**	**Parasite/qPCR Accuracy%**
CL(*n* = 20)	Skin lesion	20	0	0	0	100% (20/20) 100% (20/20)	NA	100% (20/20) 100% (20/20)
VL (*n* = 30)	Bone marrow/PBMC	20	2	1	7	91.3% (21/23) 95.6% (22/23)	NA	93.3% (28/30) 96.6% (29/30)
Non-Leish diseases^*^ (*n* = 11)	Bone marrow/PBMC	0	0	0	11	NA		
Normal (*n* = 5)	Bone marrow/PBMC	0	0	0	5	NA		
Total (*n* = 66)	Skin lesion/Bone marrow/PBMC	40	2	1	23	95.3% (41/43)97.6% (42/43)	100%	96.9% (64/66) 98.5% (65/66)

* *Including 5 with malaria, 5 with brucellosis, and 1 with dengue fever*.

### Correlation Between Parasite Load Detected by qPCR With Clinical Severity of Cutaneous and Visceral Leishmaniasis

**Figure 4:**
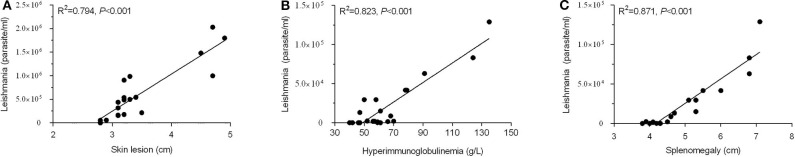
Correlation of parasite load detected by qPCR with severity of leishmaniasis. **(A)** Parasite load correlated with skin lesion diameter of CL. **(B)** Parasite load correlated with levels of serological immunoglobulin in VL. **(C)** Parasite load correlated with splenomegaly tested by ultrasonography in VL.

The established Pan-*Leishmania* qPCR assay was able to evaluate the severity of cutaneous or visceral leishmaniosis. Shown in Figure 4A, the parasite load detected by qPCR was correlated with the size of skin lesion in patients with CL. The bigger skin lesion, the higher parasite load was detected by qPCR (*R*^2^ = 0.794). The similar results were found in VL. The patients with higher level of serological immunoglobulin or bigger splenomegaly tested by ultrasonography, the common clinical signs of visceral leishmaniosis, the higher parasite burden was measured by qPCR (*R*^2^ = 0.823 and 0.871, respectively) (Figures 4B,C), indicating the mkDNA-based qPCR can be used not only for diagnosing leishmaniasis, but also for evaluating the severity of the infection.

### Assessment of the Treatment Efficacy and Prognosis of Cutaneous and Visceral Leishmaniasis by qPCR

**Figure 5:**
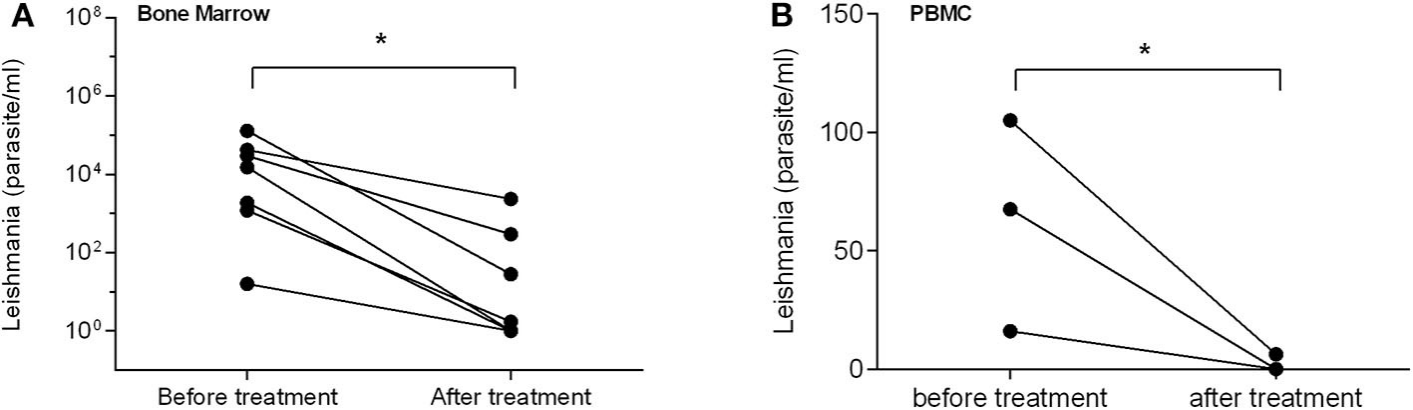
qPCR assessment of treatment efficacy of visceral leishmaniasis in samples of bone marrow **(A)** and PBMC **(B)**. Each point shows qPCR measured parasite number from individual patient before and after treatment. The bar presents median parasite number. *P < 0.05.

After being treated with sodium stibogluconate for 1–30 weeks (6–211 days), the parasite load was dramatically reduced in both bone marrow and in blood (PBMC) of patients compared with the load before treatment (*P* < 0.05, Figures 5A,B), accompanied by significantly mitigated clinical manifestations. Besides, for testing samples from VL patients, although PBMC samples were easier to acquire in clinical practice than bone marrow, there was a higher detecting rate and parasite load in bone marrow sample than blood PBMC samples by qPCR (*P* < 0.05) (Figure 5B). The results indicate that the mkDNA-based qPCR is able to monitor and assess the treatment efficacy of visceral leishmaniosis. For CL, the skin sample was not collected for qPCR detection because the skin lesion was totally healed and cured after treatment.

## Discussion

With globalization and rapid economic growth, people in developing or developed countries increase their economic activities and travel which significantly contribute to the increased world-wide transmission of some infections that are previously restricted in some specific endemic areas (Wang et al., 2019). Leishmaniasis is such a case which was prevalent in some endemic regions, but now identified in many other regions/countries with no history of the disease (Sakkas et al., 2016). The situation is further complicated by the fact that visceral and cutaneous leishmaniasis exhibit various non-specific clinical manifestations, making it hard to reliably diagnose and track the treatment. The definitive diagnosis of leishmaniasis usually replies on the identification of protozoa in the skin lesion (SL) or in blood/bone marrow (VL) that need experienced technicians or parasitologists. All these situations require a rapid, accurate and reliable diagnostic tool for leishmaniasis, especially in areas in which the disease is not endemic.

In *Leishmania* protozoa (genome size approximately 29–33 Mb), the kinetoplast organelle minicircle DNA (mkDNA) is conserved among the different *Leishmania* species (Cantacessi et al., 2015). This makes the mkDNA an attractive diagnostic biomarker for molecular detection of *Leishmania* parasite. Compared with other conserved sequences such as 18S rDNA, ITS or heat shock protein 70 KDa, the PCR based on mkDNA sequence yielded higher sensitivity and specificity than other targets, even for testing diverse clinical sample types from suspected leishmaniasis patients (Weirather et al., 2011; Real et al., 2013; Zampieri et al., 2016). In this study, we designed a new qPCR based on the mkDNA sequence regions conserved in all *Leishmania* species. The qPCR detected all three major pathogenic *L. donovani, L. infantum, and L. major* endemic or imported in China without cross-reaction with other pathogens such as *P. falciparum, T. gondii, O. tsutsugamushi, L. bacillus*, and *B. melitensis*. The pan detection of all *Leishmania* species indicates its application to detect Pan-*Leishmania* infections. The qPCR assay also has high sensitivity with ability to detect as low as one copy of mkDNA or one parasite in clinical sample.

The high sensitivity and specificity of developed Pan-*Leishmania* qPCR suggests this method could be used to better diagnose CL infected by *L. major* and VL infected mostly by *L. donovani* and *L. infantum* in China. Indeed, 20 skin lesion samples from clinically confirmed CL patients and 20 bone marrow and/or PBMC samples from clinically confirmed VL patients were tested by this new qPCR assay as all positive, with 100% sensitivity which is higher than previously reported for qPCR detection (69.23–98.53%)(Khosravi et al., 2012; El-Beshbishy et al., 2013) or microscopy smears (90–98%) (Mesa et al., 2020). We also confirmed the specificity of the assay not to cross-react with 11 clinical samples from patients with other infections with malaria, brucellosis, dengue fever and blood samples from five healthy volunteers. For nine samples from clinically cured VL patients infected with *L. infantum* (without parasite identified by microscopy or parasite culture), two of them were positively detected by qPCR in their blood samples, indicating the qPCR is a more sensitive and reliable assay than microscopy or parasite culture to diagnose leishmaniasis.

More importantly, this qPCR assay can be used not only to diagnose current infection of CL and VL patients, but also to evaluate the severity and treatment efficacy of leishmaniasis. The parasite load detected by qPCR was correlated with the severity of infection in terms of the size of lesion (CL) or the levels of serological immunoglobulin or splenomegaly tested by ultrasonography in visceral leishmaniasis. The patients with bigger skin lesion in CL and higher level of immunoglobulin or larger splenomegaly in VL, have the higher parasite load detected by qPCR. It is also noticed that the parasite load in PBMC/bone marrow of VL patients detected by this qPCR was significantly reduced after being treated with stibogluconate, accompanied with significantly mitigated clinical manifestations. The results indicate that the mkDNA-based qPCR is able to monitor and assess the treatment efficacy of visceral leishmaniosis. For CL, the skin sample was not collected for qPCR detection because the skin lesion was totally healed and cured after treatment.

The sensitive detection of *Leishmania* parasite is the key for avoiding relapse post-treatment (Martinez-Orellana et al., 2017). Previous studies have shown that a load of over 5–10 parasites/ml in the peripheral blood possibly caused clinical relapse except for other factors such as specific *Leishmania* strain, the immune status of the patient and prevalence of the disease in the region (Sudarshan et al., 2011; Sudarshan and Sundar, 2014). Thus, it is important to accurately measure the clinical therapeutic endpoint when patients are receiving therapy. Due to the high sensitivity and specificity, qPCR approach was strongly recommended by a consensus of experts to test patient sample after treatment to confirm the complete cure (Aronson et al., 2016; La Hoz and Morris, 2019; Sereno et al., 2019). Our results clearly revealed that this *Leishmania*-specific qPCR is a suitable tool not only for accurate diagnosis but also for evaluating the severity and prognosis of leishmaniasis. The qPCR assay is also useful for assess the prevalence of asymptomatic carriers of *Leishmania* in a specific population for better control of the infection in the region. Further work is needed to determine the threshold between asymptomatic and symptomatic carriage, as well as residual parasite infection level that might lead to leishmaniasis outbreak and relapse.

As a debilitating and potentially fatal neglected tropical disease, leishmaniasis needs to be well-managed and controlled in the endemic areas or non-endemic regions with potential imported cases. Since the Pan-*Leishmania* qPCR assay we developed in this study owns high sensitivity and specificity, with advantage of quick and easy operation without necessity of experienced technicians, it is a good tool for the control of leishmaniasis.

## Data Availability Statement

All datasets generated for this study are included in the article/supplementary material.

## Ethics Statement

The studies involving human participants were reviewed and approved by Ethics Committee of Beijing Friendship Hospital (Beijing, China). The patients/participants provided their written informed consent to participate in this study.

## Author Contributions

YW, XT, MH, SL, and ZW performed experiments. YW, LW, BZ, and NS analyzed data and statistics. YW and LW prepared the figures and tables. YW, NW, BZ, LW, and GY wrote the manuscript. All authors reviewed the manuscript.

## Conflict of Interest

The authors declare that the research was conducted in the absence of any commercial or financial relationships that could be construed as a potential conflict of interest.
